# Evidence of Common Genetic Overlap Between Schizophrenia and Cognition

**DOI:** 10.1093/schbul/sbv168

**Published:** 2015-12-16

**Authors:** Leon Hubbard, Katherine E. Tansey, Dheeraj Rai, Peter Jones, Stephan Ripke, Kimberly D. Chambert, Jennifer L. Moran, Steven A. McCarroll, David E. J. Linden, Michael J. Owen, Michael C. O’Donovan, James T. R. Walters, Stanley Zammit

**Affiliations:** ^1^MRC Centre for Neuropsychiatric Genetics and Genomics, Institute of Psychological Medicine and Clinical Neurosciences, School of Medicine, Cardiff University, Cardiff, UK;; ^2^School of Social and Community Medicine, University of Bristol, Bristol, UK;; ^3^Department of Psychiatry, Cambridge University, Cambridge, UK;; ^4^Stanley Center for Psychiatric Research, Broad Institute of MIT and Harvard, Cambridge, MA;; ^5^Department of Genetics, Harvard Medical School, Boston, MA

**Keywords:** schizophrenia, cognition, performance IQ, polygenic scoring, bivariate heritability

## Abstract

Cognitive impairment is a core feature of schizophrenia but there is limited understanding of the genetic relationship between cognition in the general population and schizophrenia. We examine how common variants associated with schizophrenia *en masse* contribute to childhood cognitive ability in a population-based sample, and the extent to which common genetic variants associated with childhood cognition explain variation in schizophrenia. Schizophrenia polygenic risk scores were derived from the Psychiatric Genomics Consortium (*n* = 69 516) and tested for association with IQ, attention, processing speed, working memory, problem solving, and social cognition in over 5000 children aged 8 from the Avon Longitudinal Study of Parents and Children birth cohort. Polygenic scores for these cognitive domains were tested for association with schizophrenia in a large UK schizophrenia sample (*n* = 11 853). Bivariate genome-wide complex trait analysis (GCTA) estimated the amount of shared genetic factors between schizophrenia and cognitive domains. Schizophrenia polygenic risk score was associated with lower performance IQ (*P* = .001) and lower full IQ (*P* = .013). Polygenic score for performance IQ was associated with increased risk for schizophrenia (*P* = 3.56E-04). Bivariate GCTA revealed moderate genetic correlation between schizophrenia and both performance IQ (*r*_G_ = −.379, *P* = 6.62E-05) and full IQ (*r*_G_ = −.202, *P* = 5.00E-03), with approximately 14% of the genetic component of schizophrenia shared with that for performance IQ. Our results support the presence of shared common genetic factors between schizophrenia and childhood cognitive ability. We observe a genetic relationship between schizophrenia and performance IQ but not verbal IQ or other cognitive variables, which may have implications for studies utilizing cognitive endophenotypes for psychosis.

## Introduction

Schizophrenia is a severe psychiatric disorder characterized by positive symptoms such as delusions and hallucinations, and negative symptoms such as avolition and apathy. Deficits in cognitive ability are common and are strong predictors of functional outcome,^1^ but are largely refractory to current treatments. Cognitive impairments often manifest premorbidly during childhood and adolescence,^2^ with the largest effects observed for IQ.^3,4^ These deficits persist into adulthood, typically worsen with illness onset but thereafter remain relatively static.^5^ The Measurement and Treatment Research to Improve Cognition in Schizophrenia (MATRICS)^6^ initiative identified 7 separable domains showing deficits in schizophrenia: attention, processing speed, reasoning/problem solving, social cognition, verbal learning, visual learning and working memory.

There is long-standing debate about the degree of genetic overlap between cognition in the general population and schizophrenia. The proportion of the phenotypic correlation between cognition and schizophrenia that is due to shared genetic effects was found to be substantial in a number of twin studies,^7–9^ although estimates of the genetic variance for schizophrenia that was shared with cognition varied substantially. In the only twin study that was population-based rather than ascertained by case status, and which therefore used a measure of cognition that was ascertained premorbidly, only 7% of the genetic variance for psychosis was shared with cognition,^9^ compared to 21%–56% in the other studies.^7,8^ Therefore the extent to which cognitive ability and schizophrenia share genetic aetiology remains uncertain. With recent advances in genetic technology and analytic approaches, it is now possible to investigate the underlying genetic architecture of complex traits in large cohorts of unrelated individuals.

One such approach for doing this is to use polygenic score analysis in which a score representing the additive effect of large numbers of independent (ie, in approximate linkage equilibrium) single nucleotide polymorphisms (SNPs) that are associated with 1 trait also contribute to the same or a different trait in an independent sample. Three previous studies have investigated the relationship between cognition in population samples and schizophrenia using polygenic score analysis. Increased polygenic risk of schizophrenia was weakly associated with lower general cognitive ability,^10,11^ and with a decline in cognitive ability across the lifespan.^10^ However these results have not been consistently replicated^12^ and polygenic risk scores for schizophrenia explained only a small proportion of variance in cognition (*R* ^2^ ≈ 1%). A reverse approach showed polygenic scores for general cognition (“g”) were associated with schizophrenia, but explained a small proportion of variance (*R* ^2^ < 0.5%).^11^ These studies all focused predominantly on “g” derived from different cognitive measures across multiple participating centers. To our knowledge, no studies to date have examined the genetic overlap between schizophrenia and a range of cognitive domains. This study aims to quantify the polygenic overlap between schizophrenia and individual cognitive domains and thus inform the debate about the strength of their genetic relatedness.

A second approach for investigating the underlying genetic architecture of complex traits is to use Genome-wide Complex Trait Analysis (GCTA) to estimate the proportion of phenotypic variance explained by common SNPs across the genome.^13^ It does this by first determining the degree of genetic relatedness between all individuals and then estimating the proportion of phenotypic variance explained by the genetic relatedness matrix using mixed-model analysis.^13^ The bivariate extension of GCTA allows quantification of the shared common genetic factors between 2 traits by determining the genetic variance of each trait as well as the genetic covariance, and then calculating the genetic correlation.^14^ This method has been applied previously to show significant shared common genetic aetiology between different psychiatric disorders.^15^ No previous studies have used bivariate GCTA to examine the amount of shared common genetic factors between cognition and schizophrenia.

In this study we use data from the largest schizophrenia genome-wide association study (GWAS) undertaken to date,^16^ and birth cohort data on a broad range of cognitive phenotypes measured during childhood to undertake a detailed investigation of the genetic relationship between schizophrenia and cognition using both polygenic scoring and bivariate GCTA. More specifically, we examine (1) whether common variants associated with schizophrenia *en masse* contribute to cognition in the general population at age 8, (2) the extent to which common genetic variants associated with childhood cognition *en masse* explain variation in the genetic liability to schizophrenia, and (3) the extent to which the phenotypic correlation between cognition and schizophrenia is explained by common genetic effects.

## Methods

### Samples

#### Cognition Samples.

The Avon Longitudinal Study of Parents and Children (ALSPAC) is a population-based birth cohort (http://www.alspac.bris.ac.uk) of, initially, 14 062 live births, and has been reported extensively.^17,18^

Phenotype data were collected by ALSPAC when participants were 8 years old. Individuals were administered the short form Wechsler Intelligence Scale for Children (WISC-III)^19^ (alternate items used for all subtests except the coding subtest) as well as 2 additional items taken from the Test of Everyday Attention for Children (TEACh)^20^ and the Diagnostic Analysis of Nonverbal Accuracy (DANVA).^21^ Cognitive tests were selected a priori based on their correspondence to MATRICS domains as we have previously reported.^22^ The following tasks were selected from the WISC-III, with their representative cognitive constructs in parentheses: coding (processing speed), digit span backward (working memory), and block design (problem solving). As a measure of verbal learning, we used the total number of nonwords correctly recalled from an adapted version of the Nonword Repetition Test.^23^ From the TEACh, the Sky Search task adjusted for motor speed was selected as our measure of attention. The DANVA was used to create a social cognition variable by totaling number of errors (incorrect assignment of emotions) across all 4 emotional domains. The selection of cognitive tests and corresponding domains mirrored previous work using these samples.^22^ We also used WISC IQ scores for verbal, performance and full IQ. The number of individuals with genotype and phenotype information in ALSPAC varied by specific test and ranged from 5109 to 5556 (supplementary table 2).

Further details on the ALSPAC sample including descriptive information about the cognitive variables, genotyping and imputation procedures can be found in supplementary text and supplementary tables 1 and 2.

#### Schizophrenia Samples.

Two independent schizophrenia samples were used as training datasets: the Schizophrenia Working Group of the Psychiatric Genetics Consortium (PGC-SCZ) and CLOZUK. The PGC-SCZ is a collaborative effort across multiple centers with the aim of using large sample sizes to uncover novel findings in genetic analyses. The majority of samples in PGC-SCZ have a European ancestry. Information regarding individual samples and QC can be found elsewhere.^16^ We use data from the CLOZUK sample as a replication dataset and therefore these individuals (cases and controls) were omitted from the PGC-SCZ sample for our primary analyses.

We further used CLOZUK as the testing dataset in the polygenic score analysis assessing the extent to which common genetic variants associated with childhood cognition explain variation in the genetic liability to schizophrenia, and in the bivariate GCTA. It was not possible to use the PGC-SCZ sample for this analysis given the lack of availability of genotype data and the requirement for this data in these analyses.

CLOZUK constitutes individuals with schizophrenia who were prescribed clozapine and received a clinician diagnosis of treatment resistant of schizophrenia. It has been demonstrated through a variety of different analyses that genetic risk factors for this sample are very similar to conventionally ascertained schizophrenia samples, including their frequency of CNVs,^24^ sign test for schizophrenia associated loci,^25^ and amount of common polygenic risk which aligns with other schizophrenia samples.^16^ Therefore the genetic risk factors and patterns of association indicate this is a highly valid schizophrenia sample for these types of genetic interrogations.

GWAS summary statistics were available for the complete PGC-SCZ sample minus individuals from the CLOZUK sample (termed PGC-SCZ hereafter), totaling 29 415 schizophrenia cases and 40 101 controls. The CLOZUK sample consists of 5554 cases and 6299 controls and has been described elsewhere.^16^

Additional information about the schizophrenia samples and genotyping can be found in supplementary text.

### Statistical Analysis

#### Polygenic Score Analyses.

Polygenic score methodology follows the procedure described by the schizophrenia PGC.^26^ For all analyses, SNPs were removed if they had a low minor allele frequency (MAF < 0.01), and were subsequently pruned for linkage disequilibrium using the clumping function (--clump) in PLINK^27^ removing SNPs within 500kb (--clump-kb) and *r* ^2^ > .25 (--clump-r2) with a more significantly associated SNP. We used the --score command in PLINK to calculated polygenic scores.^27^ Five *P*-value thresholds (*P*_T_ < .0001, .01, .1, .3, .5) for SNP association were used.

#### Polygenic Risk Scores for Schizophrenia.

Genotypes were tested for association with schizophrenia in the 2 independent schizophrenia case/controls samples (1) PGC-SCZ and (2) CLOZUK.^16^ Polygenic scores were calculated by summing the number of susceptibility alleles of each index SNP weighted by the logarithm of the SNP odds ratios. Polygenic risk scores for schizophrenia were calculated for each individual in the ALSPAC sample and linear regressions were used to test whether these scores were associated with performance on cognitive tasks. Consistent with previous analyses using the ALSPAC sample, no covariates were included in the polygenic analysis as ALSPAC has previously been shown to have no significant population stratification.^28^ We compared our original results against the results of 10 000 permutations where cognitive scores in ALSPAC individuals were randomly shuffled (supplementary text).

#### Polygenic Risk Scores for Cognition.

Genotypes were tested for association with the cognitive phenotypes in ALSPAC using linear regression under an additive genetic model in PLINK.^27^ No covariates were used in the analysis, as an EIGENSTRAT analyses did not reveal any significant population stratification. Analyses were run using the --standard-beta command in PLINK. Polygenic scores were calculated by summing the number of susceptibility alleles of each index SNP weighted by its coefficient of effect. Since genotype information is required in the target dataset, we used CLOZUK data for this analysis. Polygenic scores for each cognitive domain were calculated for each individual in the CLOZUK sample and logistic regressions were used to test whether the resulting scores were associated with schizophrenia case status. Analyses were covaried for 7 population principal components for the CLOZUK sample to ensure that estimates were not affected by subtle differences in population structure. We applied post hoc permutation correction for associations reaching nominal levels of significance. When applicable, we randomized case/control status in CLOZUK and repeated logistic regression analyses 10 000 times (supplementary text).

#### Bivariate GCTA.

Bivariate GCTA was used to estimate the genetic correlation between schizophrenia risk and cognitive ability by common SNPs across the genome.^14^ GCTA determines the degree of genetic relatedness between all individuals and then estimates the proportion of phenotypic variance explained by the genetic relatedness matrix using a mixed-effect model.^13^ The bivariate extension to this approach determines the genetic variance of each trait as well as the genetic covariance, and then calculates the genetic correlation (the ratio between genetic covariance and genetic variance). We applied a stringent cut-off for relatedness (proportion of shared genetic material <2.5%) and removed 1 individual from each genetically related pair.^13^ Bivariate GCTA was fitted with restricted maximum likelihood (REML) based on a linear model for the cognitive phenotypes and a disorder-model for schizophrenia with lifetime disorder risk set to 1%.^29^ A genetic relationship matrix was created for CLOZUK and ALSPAC. The genetic relationship matrix was analyzed for the genetic correlation (*r*_G_) between SNPs affecting schizophrenia case status and cognitive performance on the 6 domain tests and 3 IQ measures. The proportion of genetic variance (*r*_G_^2^) shared between schizophrenia and each cognitive test was calculated.^7,9^ As GCTA assesses the genetic relationship between individuals, a more rigorous correction for population stratification is warranted, therefore, 20 principal components were used as covariates.

## Results

### Polygenic Risk Scores for Schizophrenia: Association With Cognition

**Fig. 1.**
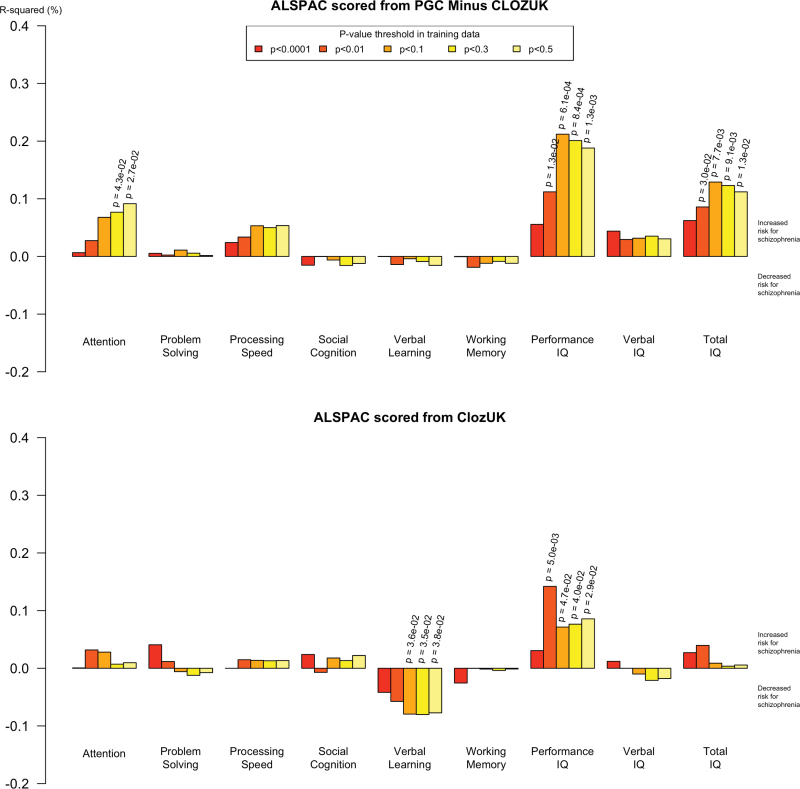
Proportion of variance in cognitive phenotypes in the Avon Longitudinal Study of Parents and Children (ALSPAC) dataset explained by schizophrenia polygenic scores. The upper panel plot uses scores derived from the Schizophrenia Working Group of the Psychiatric Genetics Consortium (PGC-SCZ) with CLOZUK omitted (PGC − CLOZUK). The lower panel plot uses scores derived from the CLOZUK sample. Two-sided *P*-values for evidence at *P* < .05 are displayed. *R* ^2^ values above 0 reflect direction of effects consistent with our hypotheses that schizophrenia polygenic risk is associated with lower cognitive scores; *R* ^2^ values below 0 reflect direction of effects opposite to those hypothesized.

We examined if polygenic scores for schizophrenia were associated with cognitive performance, with the hypothesis that the schizophrenia risk alleles *en masse* would be associated with decreased cognitive performance. Associations with performance, verbal and full IQ are presented in table 1 and figure 1, and associations with cognitive sub-domains are presented in table 2 and figure 1. Schizophrenia polygenic risk scores derived from PGC-SCZ were associated with lower performance IQ in ALSPAC at age 8 (*P* = .001; *R* ^2^ = 0.19%; *P*_T_ = .5). This result was observed at multiple training set *P*-value thresholds and replicated using schizophrenia polygenic risk scores derived from the independent CLOZUK sample (*P* = .029; *R* ^2^ = 0.09%; *P*_T_ = .5).

**Table 1.** Results From Polygenic Scores Analysis for the Global Measures of IQ

Training Dataset	Testing Dataset	*P*_T_	*N* SNPs	Performance IQ			Verbal IQ			Full IQ		
				D	*R* ^2^ (%)	*P*-value	D	*R* ^2^ (%)	*P*-value	D	*R* ^2^ (%)	*P*-value
PGC-SCZ without CLOZUK	ALSPAC cognition	.0001	1497	−	0.0556	.079	−	0.0439	.119	−	0.0621	.064
		.01	16 721	−	*0.112*	*.013*	−	0.0294	.202	−	*0.0858*	*.030*
		.1	67 032	−	*0.212*	*6.07E-04*	−	0.0316	.186	−	*0.129*	*.008*
		.3	130 553	−	*0.201*	*8.43E-04*	−	0.0352	.163	−	*0.123*	*.009*
		.5	173 932	−	*0.188*	*.001*	−	0.0305	.194	−	*0.112*	*.013*
CLOZUK SCZ	ALSPAC cognition	.0001	372	−	0.0306	.193	−	0.026	.416	−	0.011	.221
		.01	10 133	−	*0.142*	*.005*	+	0.001	.965	−	0.022	.139
		.1	58 336	−	*0.0715*	*.047*	+	0.021	.458	−	0.007	.486
		.3	127 606	−	*0.0765*	*.040*	+	0.022	.281	−	0.005	.653
		.5	177 080	−	*0.0857*	*.029*	+	0.02	.321	−	0.005	.578
ALSPAC cognition	CLOZUK SCZ	.0001	32	−	*0.028*	*.041*	+	0.003	.727	−	0.008	.359
		.01	2157	−	*0.042*	*.011*	+	0.013	.200	−	0.014	.178
		.1	19 870	−	*0.09*	*1.56E-04*	+	0.004	.632	−	0.025	.058
		.3	50 819	−	*0.084*	*2.70E-04*	+	0.004	.611	−	0.016	.140
		.5	76 137	−	*0.081*	*3.56E-04*	+	0.005	.573	−	0.013	.198

*Note*: Italicized results are for *P*-value < .05. ALSPAC, Avon Longitudinal Study of Parents and Children; PGC-SCZ, Schizophrenia Working Group of the Psychiatric Genetics Consortium; SNP, single nucleotide polymorphism. Training dataset refers to the dataset used to create the polygenic scores and testing dataset is the set tested for prediction. *P*_T_ refers to the *P*-value threshold used in the training dataset. D is the direction of effect, with negative (−) meaning increased SCZ risk is associated with decreased cognitive performance and positive (+) indicating increased SCZ risk is associated with increased cognitive performance. The number of SNPs used in the scores for the ALSPAC cognitive to CLOZUK SCZ analyses varied slightly for each cognitive domain, here we reported the average but these numbers are reported in full in supplementary table 4.

**Table 2.** Results From Polygenic Scores Analysis for the Cognitive Sub-domains

Training Dataset	Testing Dataset	*P*_T_	*N* SNPs	Attention			Problem Solving			Processing Speed			Social Cognition			Verbal Learning			Working Memory		
				D	*R* ^2^ (%)	*P*-value	D	*R* ^2^ (%)	*P*-value	D	*R* ^2^ (%)	*P*-value	D	*R* ^2^ (%)	*P*-value	D	*R* ^2^ (%)	*P*-value	D	*R* ^2^ (%)	*P*-value
PGC-SCZ without CLOZUK	ALSPAC cognition	.0001	1497	−	0.007	.555	−	0.005	.588	−	0.024	.248	+	0.015	.378	+	<0.001	.901	+	<0.001	.870
		.01	16 721	−	0.027	.228	−	0.002	.716	−	0.034	.172	+	<0.001	.910	+	0.014	.379	+	0.019	.312
		.1	67 032	−	0.068	.058	−	0.011	.436	−	0.053	.086	+	0.006	.573	+	0.004	.636	+	0.012	.423
		.3	130 553	−	*0.077*	*.043*	−	0.006	.577	−	0.050	.095	+	0.016	.370	+	0.009	.486	+	0.009	.495
		.5	173 932	−	*0.092*	*.027*	−	0.002	.767	−	0.054	.085	+	0.012	.433	+	0.015	.355	+	0.012	.422
CLOZUK SCZ	ALSPAC cognition	.0001	372	−	0.001	.856	−	0.041	.135	+	<0.001	.983	-	0.024	.269	+	0.042	.128	+	0.026	.239
		.01	10 133	−	0.032	.194	−	0.012	.422	−	0.015	.365	+	0.007	.547	+	0.057	.074	+	<0.001	.933
		.1	58 336	−	0.028	.222	+	0.006	.571	−	0.014	.384	−	0.018	.340	*+*	*0.080*	*.036*	+	0.001	.782
		.3	127 606	−	0.007	.538	+	0.012	.411	−	0.013	.396	−	0.013	.407	*+*	*0.080*	*.035*	+	0.004	.648
		.5	177 080	−	0.010	.474	+	0.008	.519	−	0.013	.389	−	0.022	.288	*+*	*0.077*	*.038*	+	0.001	.795
ALSPAC cognition	CLOZUK SCZ	.0001	32	−	0.006	.460	−	0.003	.820	+	0.003	.897	+	0.005	.523	−	0.014	.180	−	0.006	.455
		.01	2157	−	0.004	.635	+	0.008	.354	+	0.003	.827	+	0.004	.609	−	0.005	.556	+	0.004	.607
		.1	19 870	−	*0.036*	*.019*	+	0.004	.708	−	0.014	.165	−	0.005	.517	+	0.017	.120	+	0.003	.979
		.3	50 819	−	0.026	.052	−	0.003	.908	−	0.021	.081	−	0.004	.604	+	0.021	.084	−	0.003	.777
		.5	76 137	−	0.023	.070	−	0.003	.766	−	*0.029*	*.039*	−	0.005	.573	*+*	*0.029*	*.039*	−	0.003	.842

*Note*: Italicized results are for *P*-value < .05. ALSPAC, Avon Longitudinal Study of Parents and Children; PGC-SCZ, Schizophrenia Working Group of the Psychiatric Genetics Consortium; SNP, single nucleotide polymorphism. Training dataset refers to the dataset used to create the polygenic scores and testing dataset is the set tested for prediction. *P*_T_ refers to the *P*-value threshold used in the training dataset. D is the direction of effect, with negative (−) meaning increased SCZ risk is associated with decreased cognitive performance and positive (+) indicating increased SCZ risk is associated with increased cognitive performance. The number of SNPs used in the scores for the ALSPAC cognitive to CLOZUK SCZ analyses varied slightly for each cognitive domain, here we reported the average but these numbers are reported in full in supplementary table 4.

Evidence of association with other cognitive tests was less consistent; eg, increased schizophrenia polygenic risk was associated with lower full IQ when training on PGC-SCZ (*P* = .013; *R* ^2^ = 0.11%; *P*_T_ = .5) though not when training on CLOZUK. Permuted results were entirely consistent with the original regression results (supplementary table 7).

### Polygenic Risk Scores for Cognition: Association With Schizophrenia Status

**Fig. 2.**
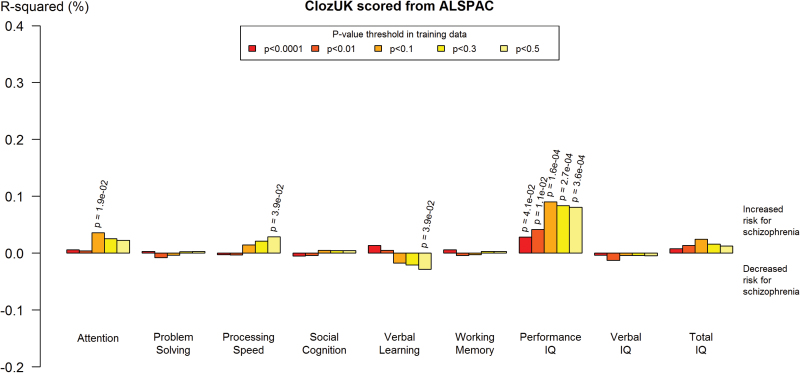
Proportion of variance in schizophrenia explained by polygenic scores of alleles associated with different cognitive phenotypes within the Avon Longitudinal Study of Parents and Children (ALSPAC) sample. The figure plots polygenic scores derived from ALSPAC cognitive phenotypes tested in the CLOZUK schizophrenia sample. Two-sided *P*-values for evidence at *P* < .05 are displayed. *R* ^2^ values above 0 reflect direction of effects consistent with our hypotheses that polygenic risk for lower cognition scoring is associated with higher schizophrenia risk; *R* ^2^ values below 0 reflect direction of effects opposite to those hypothesized.

We investigated whether genetic variants for cognition at age 8 were associated with schizophrenia, with the hypothesis that genetic variants associated with lower cognitive performance would be associated with increased risk for schizophrenia. Performance IQ polygenic score derived from ALSPAC was significantly associated with schizophrenia case status in CLOZUK (*P* = 3.56E-04; *R* ^2^ = 0.08%; *P*_T_ = .5; table 1 and figure 2). This association was seen at all training *P*-value thresholds. The direction of effect was consistent with our hypothesis. While there was limited evidence of association with processing speed, attention and verbal learning (table 2 and figure 2), these associations were weaker and less consistent across training *P*-value thresholds than those for performance IQ. Permuted results were entirely consistent with the original regression results (supplementary table 7).

To provide results for comparison, we performed split half polygenic analysis for cognition within ALSPAC and for schizophrenia within the CLOZUK. Furthermore, work completed by the Schizophrenia Working Group of Psychiatric Genomics Consortium showed that polygenic risk scores using schizophrenia risk variants derived from PGC minus CLOZUK (PGC-SCZ) reliably distinguishes between schizophrenia cases and controls in CLOZUK.^16^ At a *P*_T_ = .05 the variance explained by the PGC-SCZ polygenic score was *R* ^2^ = 0.170 in the CLOZUK sample (*P*-value < 4.26E-269 [*P*-value reported in article is 0, therefore we report it as less than the last nonzero *P*-value reported]). Further details and results are presented in supplementary tables 5 and 8 respectively.

### Bivariate GCTA

Bivariate GCTA revealed evidence of a common genetic overlap between schizophrenia and cognition. The genetic correlation (*r*_G_) was −.379 (*P* = 6.62E-05) between performance IQ and schizophrenia, and −0.202 between full IQ and schizophrenia (*P* = 5.00E-03; table 3). The negative genetic correlation indicates that alleles associated with increased schizophrenia risk are associated with poorer cognitive function. Thus approximately 14% of the genetic component for schizophrenia is shared with that for performance IQ measured at age 8 and 4% for full IQ (estimated as *r*_G_^2^). Evidence of genetic sharing was weaker for working memory and schizophrenia (*r*_G_ = −.193; *P* = .044), whilst there was no evidence to support the presence of shared genetic aetiology with schizophrenia for any of the other cognitive domains (table 3). Univariate GCTA results for each phenotype can be found in supplementary table 6.

**Table 3.** Results From the Bivariate GCTA Analysis

Schizophrenia Dataset	Cognitive Tests From ALSPAC	Schizophrenia # (Case/Control)	ALSPAC #	# SNPs	_$h_{\text{SNP}}^{2}$_ (SE) CLOZUK	_$h_{\text{SNP}}^{2}$_ (SE) Cognitive Test	*r*_G_ (SE)	*r*_G_^2^	*P*-value
CLOZUK	Attention	5336/6023	4711	424 126	0.368 (0.016)	0.006 (0.083)	1.00 (6.845)	1	.116
	Problem solving	5336/6023	4869	424 126	0.369 (0.016)	0.286 (0.081)	−.094 (0.086)	.009	.268
	Processing speed	5336/6023	4919	424 126	0.370 (0.016)	0.238 (0.082)	−.147 (0.096)	.022	.114
	Social cognition	5336/6023	4519	424 126	0.369 (0.016)	<0.001 (0.087)	1.00 (174.306)	1	.736
	***Working memory***	***5336/6023***	***4797***	***424 126***	***0.370 (0.016***)	***0.231 (0.083***)	−***.193 (0.101***)	***.037***	***.044***
	Verbal learning	5336/6023	4916	424 126	0.369 (0.016)	0.278 (0.081)	0.036 (0.087)	.001	.680
	***Performance IQ***	***5336/6023***	***4902***	***424 126***	***0.369 (0.016***)	***0.231 (0.080***)	−***.379 (0.113***)	***.144***	***6.62E-05***
	Verbal IQ	5336/6023	4905	424 126	0.370 (0.016)	0.511 (0.080)	−.067 (0.064)	.004	.296
	***Full IQ***	***5336/6023***	***4886***	***424 126***	***0.370 (0.016***)	***0.403 (0.080***)	−***.202 (0.074***)	***.041***	***5.00E-03***

*Note*: Bold and italicized results are for *P*-value < .05. $h_{\text{SNP}}^{2}$ , SNP based heritability; *r*_G_, genetic correlation; SNP, single nucleotide polymorphism. Two-sided *P*-values reported testing if *r*_G_ is different from 0.

## Discussion

We have investigated the relationship between cognition and schizophrenia using data from a large population sample and multiple independent, large schizophrenia datasets. We tested both whether polygenic scores for cognition measured at age 8 were associated with schizophrenia and if SNPs conferring risk for schizophrenia were associated with cognitive performance in children at age 8. We found a consistent, reciprocal relationship between SNPs associated with schizophrenia and SNPs associated with performance IQ using polygene scoring analysis and bivariate GCTA. There was no evidence to support genetic overlap between schizophrenia and verbal IQ, and hence weaker evidence for full IQ (as this is constructed from performance and verbal IQ). The finding that the genetic overlap between cognition and schizophrenia is strongest for performance IQ is noteworthy and should inform endophenotype research strategies particularly given previous findings that performance IQ is compromised when compared to verbal IQ in patients and their relatives.^30,31^ Little evidence was found to support genetic overlap between schizophrenia and other specific cognitive domains using either the polygenic score analysis or bivariate GCTA.

In order to quantify the genetic overlap between schizophrenia and cognition, we used the genetic correlation (*r*_G_), which indexes the correlation between genetic effects of 2 phenotypes. The genetic correlation between full IQ and schizophrenia that we observed using bivariate GCTA (*r*_G_ = −.202) was smaller than estimated through 2 twin studies that identified twin pairs on the basis of their case status and measurements of cognition post onset of schizophrenia (*r*_G_ = −.75 and *r*_G_ = −.46, respectively). It should be noted that our estimates are based only on variants tagged by common SNPs^13^ and that these comparisons are limited by differences in cognitive measures used, sample ascertainment strategies, and differing study designs. Despite this our findings align well with a population-based twin study that reported a genetic correlation between psychosis and premorbidly-assessed full IQ of −0.26.^9^ The similar association between IQ and schizophrenia across different co-relative groups reported in a recent population-based study also suggests that shared genetic effects are likely to be very modest.^32^

Our results partially replicate previous work investigating the genetic overlap between schizophrenia and cognition using polygenic score analysis that focused solely on full IQ or on “g.”^11,12^ The amount of variance in full IQ explained by schizophrenia polygenic risk in our study (*R* ^2^ ~ 0.1%) is lower than reported for “g” (*R* ^2^ ~ 0.5% to 1%) even though our training dataset was substantially larger.^11^ Our study was equivalent in terms of the size of the cognitive datasets, but all studies to date are likely to have been underpowered to accurately measure genetic effects on cognition,^12^ which might explain why results across studies are inconsistent in estimating effect sizes.^33^ It is also possible that our study differs from previous studies in terms of differences in the cognitive tests used or age at which the cognitive tests were assessed. Nevertheless, it is apparent that the magnitude of the overlapping polygenic signal between schizophrenia and cognition is modest, as measured by different studies to date.

As heritability of IQ increases throughout childhood and early adolescence,^34,35^ it may be that genetic effects are contributing to cognition to a lesser extent in our study, which assessed cognition during childhood, than in previous studies that assessed cognition during adulthood. However, GCTA estimates of common genetic factors contributing to IQ in childhood are still substantial ($h_{\text{SNP}}^{2}$ ranging from 0.22 to 0.46)^36^ and we observe similar estimates in the present study (table 3 for $h_{\text{SNP}}^{2}$ for cognitive test in ALSPAC). Furthermore, these estimates are similar to those from twin studies (*h* ^2^ = 0.41).^37^

### Implications

Our comprehensive approach to investigating common genetic architecture across cognition and schizophrenia leads us to 3 important observations. First, whilst genetic effects are shared between cognition measured at age 8 and schizophrenia this is primarily with performance IQ, implicating fluid rather than crystallized intelligence.^38^ Despite evidence of shared genetic effects between performance IQ and schizophrenia, the extent to which common SNPs explain variation in these phenotypes is modest. While there was a sizeable genetic correlation between schizophrenia and performance IQ (*r*_G_ = −.38±0.11 SE), this is smaller (though less precisely estimated) than that reported between schizophrenia and major depressive disorder (*r*_G_ = .43±0.06 SE), and substantially smaller than between schizophrenia and bipolar disorder (*r*_G_ = .68±0.04 SE), although higher than several other neuropsychiatric disorders.^15^

Second, most cognitive measures that we examined, which map onto the MATRICS domains, show little or no evidence of genetic overlap with schizophrenia. If this is replicated in other studies, it will call into question their use as endophenotypes. Thirdly the fact that cognition measured in a general population sample at age 8 shows genetic overlap with schizophrenia provides support for a genetic contribution to the neurodevelopmental nature of schizophrenia.

Endophenotypes as originally conceived were seen as aiding gene discovery for phenotypes such as schizophrenia. Our results, together with those in adult populations, suggest cognitive measures will have limited utility in this sense. A more productive approach may be to leverage the genetic overlap between cognition and schizophrenia by using cognition as a stratifier in schizophrenia discovery GWAS.^39^

It is possible that our findings of limited genetic sharing reflect the fact that we have only examined the effects of single common genotyped variants that collectively explain limited heritability or that measures used to date do not adequately capture aspects of cognition that are most relevant to the genetic aetiology of schizophrenia. For example, cognitive models of psychosis suggest that deficits in source monitoring, reasoning biases and predictive error processing may play key roles in the aetiology of psychotic experiences, and provide an explanatory link between altered biological processes and psychopathology.^40–42^ Whilst numerous studies have measures of cognitive ability related to IQ, working memory, processing speed and attention, very few studies have measures that directly capture deficits in the cognitive models of psychosis described above. If these show greater merit as endophenotypes for schizophrenia than currently assessed cognitive domains, their value will most likely be in helping to understand the mechanisms mediating genetic risk for schizophrenia as identified through large-scale collaborative genome-wide enterprises (eg, recent genome-wide evidence of association for over 100 SNPs for schizophrenia from a consortium of >35 000 cases).^16^

### Strengths and Limitations

Our study has many strengths including use of the world’s largest schizophrenia dataset, a large and relatively homogenous population based sample for assessing cognition, and use of cognitive sub-domains as well as general IQ measures. Furthermore, we assess the genetic relationship between schizophrenia and cognition using 2 different complementary approaches, polygenic analysis and bivariate GCTA. One of the main limitations of our study is that despite the use of one of the largest population-based birth cohorts worldwide with the required cognitive and genetic data, this sample may be too small to serve as a training dataset for deriving polygenic scores of cognitive phenotypes. However, to our knowledge, this is the largest homogenous collection of childhood cognitive data available currently. Furthermore, we have used only cognitive data measured during childhood and are not able to examine the possible influence of varying heritability at different ages, although our results using GCTA provide evidence that cognition at age 8 clearly has a substantial common genetic component. It is challenging to make comparisons between variance explained in “within trait” analyses (such as that presented in supplementary tables 5 and 8) and the primary cross-trait analyses we present between schizophrenia and cognition, particularly given the difference in power between samples. Such comparisons will be aided by equivalent studies conducted in very large cognitive datasets in which the phenotypic variance explained by common GWAS variants reaches a plateau as has begun to be observed in the PGC-SCZ datasets.^16^ A potential limitation is that we cannot identify individuals in the ALSPAC sample that will develop schizophrenia, and that these individuals may bias the results. However, this would apply to less than 1% of the ALSPAC sample and given the normal distribution of polygenic scores for schizophrenia and cognition it seems inconceivable that they would explain the observed results. Another limitation is that rare genetic variants are not captured by GWAS, and therefore do not contribute to our estimates; we are therefore capturing the lower bound estimate of the genetic overlap between cognition and schizophrenia. Individuals with lower cognition were also more likely to have missing genetic data (supplementary table 3), thus our estimates of the genetic overlap between schizophrenia and cognition is likely to be conservative. A final limitation is that we conducted tests at multiple *P*-value thresholds across cognitive outcomes. Whilst there is no established methodology for correcting for such correlated polygenic tests we did confirm nominally significant results with permutation. Significant associations between schizophrenia and performance IQ were observed in both polygenic score and GCTA analyses and we feel this is unlikely to have arisen due to type I error resulting from multiple testing.

In conclusion, we have undertaken a detailed examination of the common genetic architecture across cognition and schizophrenia. Our results show the relationship between schizophrenia and performance IQ is in part due to shared common genetic factors, and that this relationship is evident using a childhood cognitive sample. Our results suggest that identifying genetic factors underlying cognitive endophenotypes will capture only modest proportions of schizophrenia genetic risk. The true value of cognitive endophenotypes may lie in helping to understand the mechanisms mediating the relationship between genes and disorder.

## Supplementary Material

Supplementary material is available at http://schizophreniabulletin.oxfordjournals.org.

## Funding

UK Medical Research Council (74882); the Wellcome Trust (076467); UK Medical Research Council (MR/K004360/1 and MR/M006727/1 to D.E.J.L. and paid for K.E.T.’s salary); University of Bristol provided core support for ALSPAC. Work at the Broad Institute was funded by a philanthropic gift to the Stanley Center for Psychiatric Research. L.H. was supported by a UK Medical Research Council PhD studentship.

## Supplementary Material

Supplementary Data
