# Supplementary material for: Evidence of Common Genetic Overlap Between Schizophrenia and Cognition
Source: Schizophr Bull. 2015 Dec 16;42(3):832–42. doi: 10.1093/schbul/sbv168 (PMC4838093; doi:10.1093/schbul/sbv168)
Supplement: Supplementary Data [file supp_42_3_832__index.html]

Evidence of Common Genetic Overlap Between Schizophrenia and Cognition — Evidence of Common Genetic Overlap Between Schizophrenia and Cognition — Supplementary Data 

# Evidence of Common Genetic Overlap Between Schizophrenia and Cognition

## Supplementary Data

Data files

- Supplementary Data - Supplementary Data
