# Supplementary material for: Evidence of Common Genetic Overlap Between Schizophrenia and Cognition
Source: Schizophr Bull. 2015 Dec 16;42(3):832–42. doi: 10.1093/schbul/sbv168 (PMC4838093; doi:10.1093/schbul/sbv168)
Supplement: Supplementary Data [file supp_sbv168_Hubbard_polygene_cognition_supplementary_info_SchizBull_28september2015.doc]

# Supplementary Materials:

**Evidence of common genetic overlap between schizophrenia and cognition.**

Authors: Leon Hubbard, PhD^1*^, Katherine E. Tansey, PhD^1*^, Dheeraj Rai, PhD^2^, Peter Jones, PhD^3^, Stephan Ripke, MD^4^, Kimberly D. Chambert, MS^4^, Jennifer L. Moran, PhD^4^, Steven A. McCarroll, PhD^4,5^, David Linden, PhD^1^, Michael J. Owen, FRCPsych, PhD^1^, Michael C. O’Donovan, FRCPsych, PhD^1^, James TR Walters, PhD^1^, Stan Zammit, PhD^1,2^

[Supplementary Materials: 1](#__RefHeading___Toc431204476)

[ALSPAC sample information 1](#__RefHeading___Toc431204477)

[Description of permutation-based approach for polygenic scoring in ALSPAC 2](#__RefHeading___Toc431204478)

[Supplementary Table 1: Distribution of cognitive variables in the entire ALSPAC sample. 3](#__RefHeading___Toc431204479)

[Supplementary Table 2: Distribution of cognitive variables in only individuals with both phenotype and genotype information in the ALSPAC sample. 3](#__RefHeading___Toc431204480)

[Supplementary Table 3: t-test results comparing individuals with and without genotype data in ALSPAC sample. 3](#__RefHeading___Toc431204481)

[Supplementary Table 4: Numbers of SNPs used to generate the scores for each cognitive tests. 4](#__RefHeading___Toc431204482)

[Supplementary Table 5: Results from polygenic scores analysis within ALSPAC. 4](#__RefHeading___Toc431204483)

[Supplementary Table 6: Univariate results from GCTA for each phenotype. 5](#__RefHeading___Toc431204484)

[Supplementary Table 7: Post-hoc polygenic score regression p-values corrected using 10,000 permutations. 6](#__RefHeading___Toc431204485)

[Schizophrenia 7](#__RefHeading___Toc431204486)

[PGC-SCZ: 7](#__RefHeading___Toc431204487)

[CLOZUK: 7](#__RefHeading___Toc431204488)

[Supplementary Table 8: Results from polygenic scores analysis within CLOZUK. 9](#__RefHeading___Toc431204489)

[References 9](#__RefHeading___Toc431204490)

# ALSPAC sample information

A total of 9,912 subjects from the ALSPAC study were genotyped using the Illumina HumanHap550 quad genome-wide SNP genotyping platform (Illumina Inc., San Diego, CA, USA) by 23andMe subcontracting the Wellcome Trust Sanger Institute (Cambridge, UK) and the Laboratory Corporation of America (Burlington, NC, USA). Data were imputed using MACH 1.0.16 Markov Chain Haplotyping software and HapMap phase 2 CEPH population (HG18, release 22). Additional quality control details can be found in supplementary text. Samples underwent routine quality control. Individuals were removed for ambiguous sex (genotypic/phenotypic sex difference), abnormal heterozygosity, cryptic relatedness up to third-degree relatives using identity by descent, genotyping completeness less than 97%, and non-European ethnicity admixture detected by a multidimensional scaling analysis seeded with HapMap 2 individuals. Markers with minor allele frequency over 0.01, at least 95% complete genotyping, and passing an exact test of Hardy–Weinberg equilibrium (p>5E-07) were retained. After quality control, 8 365 unrelated individuals and 500 527 genotyped SNPs were available for analysis. EIGENSTRAT analysis revealed no additional obvious population stratification.[^3^](#_ENREF_3) Data were imputed using MACH 1.0.16 Markov Chain Haplotyping software and HapMap phase 2 CEPH population (HG18, release 22) used as a reference set.

# Description of permutation-based approach for polygenic scoring in ALSPAC

There is no consensus about correction for multiple testing in polygenic scoring analyses and typically no correction-based methods (such as false-discovery or Bonferroni) are used. However, to address potential issues with multiple testing, we applied post-hoc permutation correction for associations reaching nominal levels of significance. Permutation correction is a well-established approach in genetic association analyses.[^4^](#_ENREF_4) To calculate permutation-based p-values, we randomly shuffled the cognitive scores in ALSPAC individuals, thus breaking the link between polygenic score and phenotype. Linear regression analyses were performed on this reshuffled data set, and this was repeated 10,000 times to generate an empirical distribution of p-values. For CLOZUK analysis, case/control status was randomly shuffled, and logistic regressions were performed on this reshuffled data set, repeating it 10,000 times to generate an empirical distribution of p-values. The permuted p-value represents the proportion of times the p-values in the permuted data sets were at least as significant as the original association. This procedure was performed separately for each cognitive test and corresponding training threshold where appropriate.

# Supplementary Table *1*: Distribution of cognitive variables in the entire ALSPAC sample.

**N is the number of individuals. SD is the standard deviation, Min is the minimum observation and Max is the maximum observation.**

| **Test** | **N** | **Mean** | **SD** | **Min** | **Max** |
| --- | --- | --- | --- | --- | --- |
| Attention | 7077 | 5.07 | 1.44 | -0.35 | 10.98 |
| Problem Solving | 7330 | 32.20 | 12.51 | 0 | 68 |
| Processing Speed | 7406 | 34.49 | 7.30 | 0 | 64 |
| Social Cognition | 6820 | 4.60 | 2.79 | 0 | 22 |
| Working Memory | 7227 | 3.52 | 0.83 | 0 | 7 |
| Verbal Learning | 7367 | 7.23 | 2.51 | 0 | 12 |
| Performance IQ | 7377 | 99.46 | 17.12 | 46 | 151 |
| Verbal IQ | 7385 | 106.96 | 16.80 | 46 | 155 |
| Total IQ | 7354 | 103.97 | 16.54 | 45 | 151 |

# Supplementary Table 2: Distribution of cognitive variables in only individuals with both phenotype and genotype information in the ALSPAC sample.

**N is the number of individuals. SD is the standard deviation, Min is the minimum observation and Max is the maximum observation.**

| **Test** | **N** | **Mean** | **SD** | **Min** | **Max** |
| --- | --- | --- | --- | --- | --- |
| Attention | 5318 | 5.07 | 1.43 | 0.60 | 10.94 |
| Problem Solving | 5499 | 32.38 | 12.46 | 0 | 68 |
| Processing Speed | 5556 | 34.51 | 7.29 | 0 | 64 |
| Social Cognition | 5109 | 4.59 | 2.77 | 0 | 22 |
| Working Memory | 5420 | 3.54 | 0.84 | 0 | 7 |
| Verbal Learning | 5552 | 7.26 | 2.51 | 0 | 12 |
| Performance IQ | 5535 | 100.05 | 16.96 | 46 | 147 |
| Verbal IQ | 5540 | 107.81 | 16.75 | 50 | 155 |
| Total IQ | 5517 | 104.79 | 16.41 | 45 | 151 |

# Supplementary Table *3*: t-test results comparing individuals with and without genotype data in ALSPAC sample.

**Mean ungenotyped is the mean of that test for the ungenotyped individuals, mean genotyped is the mean of that test for the genotype individuals, t is the t statistic, df is the degrees of freedom, p-values reported are two-tailed.**

| **Test** | **Mean ungenotyped** | **Mean genotyped** | **t** | **df** | **p-value** |
| --- | --- | --- | --- | --- | --- |
| Attention | 5.07 | 5.07 | -0.15 | 7075 | 0.881 |
| Problem Solving | 31.64 | 32.38 | -2.21 | 7328 | 0.027 |
| Processing Speed | 34.41 | 34.51 | -0.52 | 7404 | 0.602 |
| Social Cognition | 4.63 | 4.59 | 0.41 | 6818 | 0.684 |
| Working Memory | 3.48 | 3.54 | -2.70 | 7225 | 0.007 |
| Verbal Learning | 7.14 | 7.26 | -1.82 | 7365 | 0.069 |
| Performance IQ | 97.68 | 100.05 | -5.16 | 7375 | 2.54E-07 |
| Verbal IQ | 104.38 | 107.81 | -7.62 | 7383 | 2.91E-14 |
| Total IQ | 101.50 | 104.79 | -7.43 | 7352 | 1.25E-13 |

# Supplementary Table *4*: Numbers of SNPs used to generate the scores for each cognitive tests.

P_T_ refers to the p-value threshold used in the training dataset.

| Cognitive Test | P_T_ | | | | |
| --- | --- | --- | --- | --- | --- |
|  | 0.0001 | 0.01 | 0.1 | 0.3 | 0.5 |
| Attention | 38 | 2391 | 19289 | 50379 | 75667 |
| Problem Solving | 39 | 2529 | 20132 | 50806 | 76148 |
| Processing Speed | 31 | 2499 | 19623 | 50618 | 75951 |
| Social Cognition | 28 | 2279 | 19082 | 50242 | 75860 |
| Working Memory | 25 | 2470 | 19793 | 50502 | 75607 |
| Verbal Learning | 27 | 2534 | 20089 | 51055 | 76331 |
| Performance IQ | 32 | 2500 | 19930 | 50868 | 76368 |
| Verbal IQ | 35 | 2739 | 20526 | 51657 | 76774 |
| Total IQ | 31 | 2710 | 20363 | 51241 | 76526 |

# Supplementary Table *5*: Results from polygenic scores analysis within ALSPAC.

**The ALSPAC sample was randomly split in half ensuring that groups did not differ based on gender or cognitive score. Training dataset refers to the dataset used to create the polygenic scores and testing dataset is the set tested for prediction. P_T_ refers to the p-value threshold used in the training dataset. R^2^ and p-values are outputs from the linear regression where cognitive score was dependent variable and polygenic score for cognition was the independent variable.**

| **Training**  **Dataset** | **Testing**  **Dataset** | ***P_T_*** | **N SNPs in**  **Training Set** | **R^2^** | **P-value** |
| --- | --- | --- | --- | --- | --- |
| **ALSPAC Attention**  **(n=2592)** | **ALSPAC Attention**  **(n=2726)** | 0.0001 | 35 | 0.0000 | 9.92E-01 |
|  |  | 0.01 | 1638 | 0.0003 | 3.56E-01 |
|  |  | 0.1 | 13513 | 0.0005 | 2.38E-01 |
|  |  | 0.2 | 24360 | 0.0000 | 8.24E-01 |
|  |  | 0.3 | 34505 | 0.0000 | 9.44E-01 |
|  |  | 0.4 | 43840 | 0.0000 | 8.56E-01 |
|  |  | 0.5 | 52423 | 0.0000 | 7.79E-01 |
| **ALSPAC Problem Solving**  **(n=2700)** | **ALSPAC Problem Solving**  **(n=2799)** | 0.0001 | 28 | 0.0012 | 7.19E-02 |
|  |  | 0.01 | 1683 | 0.0021 | 1.42E-02 |
|  |  | 0.1 | 13499 | 0.0046 | 3.51E-04 |
|  |  | 0.2 | 24700 | 0.0042 | 6.25E-04 |
|  |  | 0.3 | 35114 | 0.0056 | 7.64E-05 |
|  |  | 0.4 | 44516 | 0.0060 | 3.81E-05 |
|  |  | 0.5 | 53005 | 0.0053 | 1.11E-04 |
| **ALSPAC Processing Speed**  **(n=2731)** | **ALSPAC Processing Speed**  **(n=2825)** | 0.0001 | 32 | 0.0005 | 2.44E-01 |
|  |  | 0.01 | 1731 | 0.0000 | 9.66E-01 |
|  |  | 0.1 | 13295 | 0.0000 | 8.97E-01 |
|  |  | 0.2 | 24547 | 0.0001 | 5.33E-01 |
|  |  | 0.3 | 34660 | 0.0001 | 5.89E-01 |
|  |  | 0.4 | 44095 | 0.0002 | 4.20E-01 |
|  |  | 0.5 | 52782 | 0.0003 | 3.75E-01 |
| **ALSPAC Social Cognition**  **(n=2511)** | **ALSPAC Social Cognition**  **(n=2598)** | 0.0001 | 37 | 0.0003 | 3.56E-01 |
|  |  | 0.01 | 1652 | 0.0001 | 5.45E-01 |
|  |  | 0.1 | 13157 | 0.0001 | 5.69E-01 |
|  |  | 0.2 | 24374 | 0.0006 | 1.97E-01 |
|  |  | 0.3 | 34519 | 0.0004 | 2.88E-01 |
|  |  | 0.4 | 43968 | 0.0003 | 3.58E-01 |
|  |  | 0.5 | 52503 | 0.0003 | 4.04E-01 |
| **ALSPAC Verbal Learning**  **(n=2740)** | **ALSPAC Verbal Learning**  **(n=2812)** | 0.0001 | 28 | 0.0005 | 2.20E-01 |
|  |  | 0.01 | 1706 | 0.0006 | 2.11E-01 |
|  |  | 0.1 | 13597 | 0.0023 | 1.11E-02 |
|  |  | 0.2 | 24759 | 0.0016 | 3.41E-02 |
|  |  | 0.3 | 34984 | 0.0019 | 1.94E-02 |
|  |  | 0.4 | 44496 | 0.0028 | 5.01E-03 |
|  |  | 0.5 | 53230 | 0.0032 | 2.67E-03 |
| **ALSPAC Working Memory**  **(n=2659)** | **ALSPAC Working Memory**  **(n=2761)** | 0.0001 | 35 | 0.0000 | 8.87E-01 |
|  |  | 0.01 | 1718 | 0.0002 | 4.14E-01 |
|  |  | 0.1 | 13618 | 0.0011 | 7.88E-02 |
|  |  | 0.2 | 24598 | 0.0014 | 5.15E-02 |
|  |  | 0.3 | 34825 | 0.0010 | 9.46E-02 |
|  |  | 0.4 | 44174 | 0.0010 | 1.05E-01 |
|  |  | 0.5 | 53036 | 0.0011 | 8.54E-02 |
| **ALSPAC Performance IQ**  **(n=2715)** | **ALSPAC Performance IQ**  **(n=2820)** | 0.0001 | 26 | 0.0033 | 2.48E-03 |
|  |  | 0.01 | 1752 | 0.0027 | 5.83E-03 |
|  |  | 0.1 | 13577 | 0.0027 | 5.97E-03 |
|  |  | 0.2 | 24779 | 0.0026 | 6.65E-03 |
|  |  | 0.3 | 35211 | 0.0024 | 9.81E-03 |
|  |  | 0.4 | 44625 | 0.0004 | 2.65E-01 |
|  |  | 0.5 | 53097 | 0.0003 | 3.47E-01 |
| **ALSPAC Verbal IQ**  **(n=2720)** | **ALSPAC Verbal IQ**  **(n=2820)** | 0.0001 | 52 | 0.0004 | 3.12E-01 |
|  |  | 0.01 | 1893 | 0.0017 | 2.96E-02 |
|  |  | 0.1 | 13729 | 0.0099 | 1.25E-07 |
|  |  | 0.2 | 25085 | 0.0108 | 2.96E-08 |
|  |  | 0.3 | 35268 | 0.0119 | 6.53E-09 |
|  |  | 0.4 | 44669 | 0.0124 | 3.21E-09 |
|  |  | 0.5 | 53278 | 0.0128 | 1.78E-09 |
| **ALSPAC Full IQ**  **(n=2706)** | **ALSPAC Full IQ**  **(n=2811)** | 0.0001 | 29 | 0.0000 | 7.88E-01 |
|  |  | 0.01 | 1758 | 0.0031 | 3.23E-03 |
|  |  | 0.1 | 13601 | 0.0095 | 2.37E-07 |
|  |  | 0.2 | 25010 | 0.0121 | 4.94E-09 |
|  |  | 0.3 | 35066 | 0.0129 | 1.45E-09 |
|  |  | 0.4 | 44430 | 0.0117 | 8.78E-09 |
|  |  | 0.5 | 53133 | 0.0122 | 4.31E-09 |

# Supplementary Table *6*: Univariate results from GCTA for each phenotype.

N is the number of individuals. # SNP is the number of SNPs used to generate the genetic relationships matrix. *h^2^_SNP_* is the amount of phenotypic variance explained by common genetic variance. SE is the standard error for *h^2^_SNP_*. P-values reported are one-sided.

| Phenotype | N | # SNPs | *h^2^_SNP_* | SE | p-value |
| --- | --- | --- | --- | --- | --- |
| Attention | 5318 | 487069 | 0.060 | 0.075 | 0.2 |
| Problem Solving | 5499 | 487069 | 0.407 | 0.073 | 5.00E-09 |
| Processing Speed | 5556 | 487069 | 0.242 | 0.074 | 6.00E-04 |
| Social Cognition | 5109 | 487069 | <0.001 | 0.076 | 0.5 |
| Working Memory | 5420 | 487069 | 0.218 | 0.073 | 1.00E-03 |
| Verbal Learning | 5552 | 487069 | 0.372 | 0.072 | 5.00E-08 |
| Performance IQ | 5535 | 487069 | 0.338 | 0.072 | 1.00E-06 |
| Verbal IQ | 5540 | 487069 | 0.605 | 0.071 | 4.00E-19 |
| Total IQ | 5517 | 487069 | 0.519 | 0.071 | 3.00E-14 |
| CLOZUK SCZ | 11466 | 424126 | 0.379 | 0.015 | 0 |

# Supplementary Table 7: Post-hoc polygenic score regression p-values corrected using 10,000 permutations.

P_T_ refers to the p-value threshold used in the training dataset. P-value (original) corresponds to non-corrected results. P-value (permuted) is the p-value after permutation correction. Results shown reached nominal levels of significance (p<0.05) in the original analyses (reported in main text).

| **Training**  **Dataset** | **Testing**  **Dataset** | ***P_T_*** | **Performance IQ** | | **Full IQ** | | **Attention** | | **Processing Speed** | | **Verbal Learning** | |
| --- | --- | --- | --- | --- | --- | --- | --- | --- | --- | --- | --- | --- |
|  |  |  | **p-value (original)** | **p-value (permuted)** | **p-value (original)** | **p-value (permuted)** | **p-value** | **p-value (permuted)** | **p-value** | **p-value (permuted)** | **p-value (original)** | **p-value (permuted)** |
| **PGC**  **SCZ Without CLOZUK** | **ALSPAC**  **Cognition** | 0.01 | ***0.013*** | ***0.013*** | ***0.030*** | ***0.028*** | - | - | - | - | - | - |
|  |  | 0.1 | ***6.07E-04*** | ***4.00E-04*** | ***0.008*** | ***0.007*** | - | - | - | - | - | - |
|  |  | 0.3 | ***8.43E-04*** | ***8.00E-04*** | ***0.009*** | ***0.010*** | ***0.043*** | ***0.046*** | ***-*** | ***-*** | ***-*** | ***-*** |
|  |  | 0.5 | ***0.001*** | ***0.001*** | ***0.013*** | ***0.011*** | ***0.027*** | ***0.029*** | ***-*** | ***-*** | ***-*** | ***-*** |
|  |  |  |  |  |  |  |  |  |  |  |  |  |
| **CLOZUK**  **SCZ** | **ALSPAC**  **Cognition** | 0.01 | ***0.005*** | ***0.005*** | ***0.005*** | ***0.005*** | - | - | - | - | - | - |
|  |  | 0.1 | ***0.047*** | ***0.043*** | ***0.047*** | ***0.043*** | ***-*** | ***-*** | ***-*** | ***-*** | ***0.036*** | ***0.034*** |
|  |  | 0.3 | ***0.040*** | ***0.041*** | ***0.040*** | ***0.041*** | ***-*** | ***-*** | ***-*** | ***-*** | ***0.035*** | ***0.032*** |
|  |  | 0.5 | ***0.029*** | ***0.029*** | ***0.029*** | ***0.029*** | ***-*** | ***-*** | ***-*** | ***-*** | ***0.038*** | ***0.039*** |
|  |  |  |  |  |  |  |  |  |  |  |  |  |
| **ALSPAC Cognition** | **CLOZUK SCZ** | 0.0001 | ***0.041*** | ***0.041*** | - | - | - | - | - | - | - | - |
|  |  | 0.01 | ***0.011*** | ***0.012*** | - | - | ***-*** | ***-*** | ***-*** | ***-*** | - | - |
|  |  | 0.1 | ***1.56E-04*** | ***2.00E-04*** | ***-*** | - | ***0.019*** | ***0.019*** | ***-*** | ***-*** | - | - |
|  |  | 0.3 | ***2.70E-04*** | ***5.00E-04*** | - | - | ***-*** | ***-*** | ***-*** | ***-*** | - | - |
|  |  | 0.5 | ***3.56E-04*** | ***3.00E-04*** | - | - | - | - | ***0.039*** | ***0.042*** | ***0.039*** | ***0.042*** |

# Schizophrenia

## PGC-SCZ:

Information for the individual sample recruitment, ascertainment and diagnoses for all samples used in the PGC can be found in the supplementary data of the original paper.[^5^](#_ENREF_5) Briefly, all cases included in the analysis had a diagnosis of schizophrenia or schizoaffective disorder. Population matched controls were available for all samples. Data used in this manuscript did not include the CLOZUK samples. As part of the Schizophrenia Working Group of the Psychiatric Genomics Consortium (PGC-SCZ) analytic pipeline, samples underwent routine quality control using PLINK[^6^](#_ENREF_6) and were imputed using the pre-phasing/imputation stepwise approach implemented in IMPUTE2/SHAPEIT and 1000Genomes (August 2012, release “v3.macGT1”) as the reference dataset.

Cross validation analysis for schizophrenia polygenic risk scores were completed as part of the Schizophrenia Working Group of the Psychiatric Genomics Consortium, which showed polygenic risk scores using schizophrenia risk variants (derived from PGC minus CLOZUK) reliably distinguishes between schizophrenia cases and controls in CLOZUK.[^5^](#_ENREF_5) At a P_T_ of 0.05 the variance explained by the PGC-CLOZUK polygenic score was R^2^= 0.1698 in the CLOZUK sample (p value<4.26E-269 (p-value reported in paper is 0, therefore report it as less than the last non-zero p-value reported)). For the full results of this analysis we direct readers to the original paper and to the supplementary tables (CLOZUK referred to as ‘noclo_clo’): <http://www.nature.com/nature/journal/v511/n7510/fig_tab/nature13595_SF6.html> and <http://www.nature.com/nature/journal/v511/n7510/extref/nature13595-s5.xls>

## CLOZUK:

Ascertainment of samples has been described previously.[^5^](#_ENREF_5) Briefly, cases were ascertained through facilitation with Novartis, the manufacturer of a proprietary form of clozapine (Clozaril), and consisted of individuals with treatment-resistant schizophrenia according to the clozapine registration forms completed by treating psychiatrists. The controls were from Wellcome Trust Case Control Consortium 2 (WTCCC2) (National Blood Service (NBS) and 1958 Birth Cohort). Samples were genotyped on the Illumina HumanOmniExpressExome 8v1 and Illumina HumanOmniExpress-12v1 (Illumina Inc., San Diego, CA, USA). SNP quality control, and imputation were performed by the PGC Statistical Analysis Group.[^5^](#_ENREF_5) Briefly, quality control parameters were: SNP missingness<5% (before sample removal); subject missingness<2%; autosomal heterozygosity deviation<0.2); SNP missingness<2% (after sample removal); difference in SNP missingness between cases and controls<2%; and SNP Hardy-Weinberg equilibrium (P>1E−06 in controls or P>1E−10 in cases). Genotype imputation was performed using IMPUTE2 / SHAPEIT. The imputation reference set consisted of 2,186 phased haplotypes from the 1000 Genomes Project (August 2012, 30,069,288 variants, release “v3.macGT1”).

After QC, summary statistics were available for 5554 cases and 6299 controls.

We performed within-sample validation using a natural split within the CLOZUK sample. Our training schizophrenia dataset was genotyped on the Illumina HumanOmniExpressExome 8v1 (called CLOZUK-training, totalling 3446 cases and 4825 controls) and out target dataset was genotyped on the Illumina HumanOmniExpress-12v1 (called CLOZUK-target, totalling 2108 cases and 2014 controls) (Illumina Inc.). Polygenic scores were derived as described in the methods section. We used a logistic regression model to investigate the strength of association between schizophrenia case/control status and schizophrenia polygenic risk scores in CLOZUK-target adjusting for 10 principle components measuring population stratification. Concordant with previous studies, we show schizophrenia cases have significantly higher polygenic risk scores for schizophrenia than healthy controls.

# Supplementary Table 8: Results from polygenic scores analysis within CLOZUK.

Training dataset refers to the dataset used to create the polygenic scores and testing dataset is the target set to which the polygenic score is applied. P_T_ refers to the p-value threshold used in the training dataset. P-values are from a logistic regression with case/control status as the dependent variable. Nagelkerke R^2^ represents the variance of schizophrenia case/control status explained by schizophrenia polygenic score and all covariates, minus the variance of case/control status explained by just the first 10 principle components.

| **Training**  **Dataset** | **Testing**  **Dataset** | ***P_T_*** | **N SNPs in**  **Training Set** | **Nagelkerke R^2^** | **P-value** |
| --- | --- | --- | --- | --- | --- |
| **CLOZUK-training**  **(3446 cases &**  **4285 controls** | **CLOZUK-target**  **(2108 cases &**  **2014 controls** | 0.00001 | 21 | 0.008 | 3.41E-07 |
|  |  | 0.0001 | 91 | 0.014 | 1.29E-11 |
|  |  | 0.001 | 554 | 0.023 | 2.47E-18 |
|  |  | 0.01 | 3521 | 0.055 | 8.18E-41 |
|  |  | 0.05 | 13030 | 0.075 | 9.00E-54 |
|  |  | 0.1 | 23319 | 0.075 | 7.35E-54 |
|  |  | 0.2 | 41230 | 0.083 | 2.50E-59 |
|  |  | 0.3 | 56859 | 0.086 | 7.89E-61 |
|  |  | 0.4 | 70835 | 0.086 | 6.96E-61 |
|  |  | 0.5 | 83529 | 0.089 | 5.69E-63 |

# References

**1.** Li Y, Willer C, Sanna S, Abecasis G. Genotype imputation. *Annu Rev Genomics Hum Genet* 2009;10:387-406.

**2.** Li Y, Willer CJ, Ding J, Scheet P, Abecasis GR. MaCH: using sequence and genotype data to estimate haplotypes and unobserved genotypes. *Genet Epidemiol* Dec 2010;34(8):816-834.

**3.** Price AL, Patterson NJ, Plenge RM, Weinblatt ME, Shadick NA, Reich D. Principal components analysis corrects for stratification in genome-wide association studies. *NatGenet* 2006;38(8):904-909.

**4.** Sham PC, Purcell SM. Statistical power and significance testing in large-scale genetic studies. *Nature reviews Genetics* May 2014;15(5):335-346.

**5.** Consortium SWGotPG. Biological insights from 108 schizophrenia-associated genetic loci. *Nature* Jul 24 2014;511(7510):421-427.

**6.** Purcell S, Neale B, Todd-Brown K, et al. PLINK: a tool set for whole-genome association and population-based linkage analyses. *AmJHumGenet* 2007;81(3):559-575.

**7.** Howie B, Marchini J, Stephens M. Genotype imputation with thousands of genomes. *G3 (Bethesda)* Nov 2011;1(6):457-470.

**8.** Delaneau O, Marchini J, Zagury JF. A linear complexity phasing method for thousands of genomes. *Nat Methods* Feb 2012;9(2):179-181.
